# Experiences of pregnant women with a third trimester routine ultrasound – a qualitative study

**DOI:** 10.1186/s12884-019-2470-9

**Published:** 2019-09-02

**Authors:** Myrte Westerneng, Mariëlle Diepeveen, Anke B. Witteveen, Marjan J. Westerman, Henriette E. van der Horst, Anneloes L. van Baar, Ank de Jonge

**Affiliations:** 1 Amsterdam UMC, Vrije Universiteit Amsterdam, Midwifery Science, AVAG, Amsterdam Public Health research institute, P.O. Box 7057, 1007 MB Amsterdam, The Netherlands; 2 Amsterdam UMC, Vrije Universiteit Amsterdam, Department of Medical Humanities and the Amsterdam Public Health research institute, Amsterdam, The Netherlands; 3 Department of Methodology and Statistics, Institute of Health Sciences, Faculty of Earth and Life Sciences, VU University, Amsterdam, The Netherlands; 4 Amsterdam UMC, Vrije Universiteit Amsterdam, Department of General Practice and Elderly Care Medicine, Amsterdam Public Health research institute, Amsterdam, The Netherlands; 5 Child and adolescent studies, Utrecht University, Utrecht, The Netherlands

**Keywords:** Pregnancy, Ultrasound, Primary care, Maternal bonding, Pregnancy anxiety, Qualitative studies

## Abstract

**Background:**

Studies showed that pregnant women generally value routine ultrasounds in the first two trimesters because these provide reassurance and a chance to see their unborn baby. This, in turn, might help to decrease maternal anxiety levels and increase the bond with the baby. However, it is unclear whether pregnant women hold the same positive views about a third trimester routine ultrasound, which is increasingly being used in the Netherlands as a screening tool to monitor fetal growth. The aim of this study was to explore pregnant women’s experiences with a third trimester routine ultrasound.

**Methods:**

We held semi-structured interviews with fifteen low-risk pregnant women who received a third trimester routine ultrasound in the context of the Dutch IUGR RIsk Selection (IRIS) study. The IRIS study is a nationwide cluster randomized controlled trial carried out among more than 13,000 women to examine the effectiveness of a third trimester routine ultrasound to monitor fetal growth. For the interviews, participants were purposively selected based on parity, age, ethnicity, and educational level. We performed thematic content analysis using MAXQDA.

**Results:**

Most pregnant women appreciated a third trimester routine ultrasound because it provided them confirmation that their baby was fine and an extra opportunity to see their baby. At the same time they expressed that they already felt confident about the health of their baby, and did not feel that their bond with their baby had increased after the third trimester ultrasound. Women also reported that they were getting used to routine ultrasounds throughout their pregnancy, and that this increased their need for another one.

**Conclusions:**

Pregnant women seem to appreciate a third trimester routine ultrasound, but it does not seem to reduce anxiety or to improve bonding with their baby. Women’s appreciation of a third trimester routine ultrasound might arise from getting used to routine ultrasounds throughout pregnancy. We recommend to examine the psychological impact of third trimester routine ultrasounds in future studies. Results should be taken into consideration when balancing the gains, which are as yet not clear, of introducing a third trimester routine ultrasound against unwanted side effects and costs.

## Background

Over the last two decades, routine ultrasound scans have become common practice in midwifery care in most Western countries [1]. Aside from a dating scan which generally takes place in the first trimester, many countries routinely offer women a fetal anomaly scan around 20 weeks gestation. Screening for Down syndrome, which usually consists of an ultrasound around 12 weeks gestation and a blood test, is being offered by many Western countries as well [2]. The recent introduction of non-invasive prenatal testing, however, might soon decrease the number of ultrasounds performed to screen for Down Syndrome [3]. Nevertheless, given that the uptake for routine ultrasounds is high [4–8], most women in Western countries receive at least two routine ultrasounds during pregnancy. In the Netherlands, like in most Western countries, third trimester ultrasounds are not part of the national policy for prenatal care [2]. However, despite inconclusive evidence about their clinical effectiveness [9], third trimester ultrasound scans are being increasingly used in the Netherlands to monitor fetal growth.

Besides screening for abnormalities in the development of the baby, routine ultrasound scans offer women a chance to see a real-time, moving image of their baby. Both pregnant women and professionals foster the idea that this strengthens the bond between a pregnant woman and her baby and gives the mother reassurance, which might help to reduce maternal anxiety levels [1, 10]. The Dutch health council mentioned this consideration in their advice to offer all pregnant women the option to screen for Down-syndrome and fetal anomalies [8]. Some researchers and professionals, however, are doubtful about the psychological benefits of routine ultrasounds. They suggest that offering routine ultrasounds without a sound medical underpinning contributes to the ‘medicalization’ of pregnancy which emphasises pregnancy-related risks, which in turn might lead to more fears and worries among pregnant women [10–12].

Most studies exploring the experiences of pregnant women with routine ultrasounds have focused on routine ultrasounds in the first two trimesters [13–16]. A review of 25 studies across a variety of countries by Garcia et al. [1] reveals that women in general hold very positive views on first and second trimester ultrasounds. Worries about what the scan might show, or that the scan may be harmful for them or the baby, are expressed by a minority of women. According to this review, a frequently mentioned reason why women value ultrasound scans, is getting reassurance, and furthermore reasons that are related to the bonding process are mentioned: meeting the baby, and feeling that their pregnancy becomes more real [17]. Some women also mention involvement of the partner in the pregnancy as an important positive consequence of routine ultrasound [17, 18]. The positive effect of ultrasounds on maternal-fetal bonding has been confirmed by some studies [19, 20], while the role of routine ultrasounds in giving women reassurance is less conclusive [21, 22]. Moreover, some argue that the reassurance that is attributed to routine ultrasounds should be interpreted with caution. A study by Harpel [23], for example, showed that women did not experience much anxiety during pregnancy until shortly before the routine ultrasound, which suggests that ‘reassurance’ happens because of anxiety created by the anticipation of the ultrasound itself. A more recent study by Thomas et al., which examined the role of ultrasounds in providing reassurance, showed that ultrasounds might play a different role in the later stages of pregnancy. While women in the first trimester expressed a need for reassurance because of specific worries, women in the second and third trimester expressed a more general need to know that their baby was fine [24].

In this paper, we focus on the experiences of pregnant women with a third trimester routine ultrasound, since research on this topic is scarce and findings from studies focusing on ultrasounds in the first two trimesters might not be generalizable to the third trimester. For example, women in the third trimester might already be more confident and feel a stronger bond with their baby, maybe also due to ultrasound scans in the first two trimesters and the presence of fetal movements. Evaluating the experiences of pregnant women with third trimester routine ultrasounds contributes to knowledge about the value of adding a third trimester routine ultrasound, from a psychological perspective, to current obstetric care. We addressed the following research question: How do low-risk pregnant women experience a third trimester routine ultrasound?

## Methods

### Study design

A qualitative study design was used to allow in-depth exploration of experiences of low-risk pregnant women who received a third trimester routine ultrasound in the context of the Dutch IUGR RIsk Selection (IRIS) study [25]. The IRIS study is a nationwide study carried out in 60 midwifery practices to evaluate the (cost-)effectiveness of the third trimester routine ultrasound.

### Sample and recruitment

Women between 20 and 24 weeks gestation with uncomplicated singleton pregnancies in midwife-led care were asked to participate in the IRIS study by their midwife between February 2015 and February 2016. At the start of the study, all midwifery practices started in the control condition in which they offered standard care (serial fundal height measurements and ultrasonography only if clinically indicated). Every 3 months, 20 practices were randomized to the intervention condition in which they offered, in addition to the clinically indicated ultrasounds, two routine ultrasounds; one between 28 and 30 weeks gestation and one between 34 and 36 weeks gestation.

More than 13,000 pregnant women were included in the IRIS study, of which more than 7000 women were offered two routine ultrasounds in the third trimester. For this study, we selected pregnant women from a list of IRIS study participants who had received at least one third trimester routine ultrasound and who had sufficient command of the Dutch language. To obtain a varied sample, women also were purposively selected based on parity, age, ethnicity and educational level. All women were invited by a telephone call in which the aim of the study and a brief overview of the content of the interview was given. Women were free to choose where they wanted the interview to take place, as long as it was a quiet environment. Reasons not to participate were time constraints or a lack of interest. After 13 interviews were carried out, we decided to interview two more women who had a suspicion of growth-restriction of their fetus based on the third trimester routine ultrasound. The reason to do this was that this only had occurred once in the initially selected sample. We also wanted to be sure that data saturation was reached and no new information emerged [26]. Of the 19 women that were approached, 15 (79%) agreed to participate. Interviews took place between December 2015 and May 2016.

### Interviews

The authors formulated an initial topic-list based on relevant literature [1, 19, 20, 27, 28]. This resulted in three main topics: (1) Feelings during pregnancy (which addressed both feelings related and unrelated to pregnancy, and the bond experienced with the baby), (2) experiences with a third trimester routine ultrasound (the ultrasound procedure itself, but also the period prior to and the period after the third trimester routine ultrasound), and (3) the possible influence of a third trimester routine ultrasound on feelings experienced during pregnancy. For every topic, specific questions were formulated (see Table 2 in Appendix). One of the authors (MW), who carried out all the interviews, first conducted a pilot interview to become familiar with the topic-list and to test the questions. Minimal changes were made. Prior to each interview, MW gave a short explanation about the interview procedure and the global content of the interview. During the interviews, the interviewer gave respondents the opportunity to discuss subjects that were not part of the initial topic-list, which provided a chance to explore new paths that evolved during an interview. Based on the first three interviews, some questions were added to the initial topic-list. For example, the idea of *not* getting a third trimester routine ultrasound appeared to be a relevant topic. Also, we added a question about which people accompanied women during the third trimester routine ultrasound. All respondents, except one, were interviewed at their homes. One participant preferred to be interviewed at work in a separate meeting room. Interviews lasted on average 37 min (range 21–65 min) and were audiotaped and transcribed verbatim by student assistants who received instructions from the interviewer.

### Data analysis

Two researchers carried out the data analysis: MW and MD (both female PhD candidates). To get a sense of the interviews as a whole, MW first summarized the interviews, after which MW and MD discussed the summaries. Next, these two researchers performed a thematic content analysis of the interviews which consists of open coding (giving labels to single constructs), axial coding (categorizing the labels) and selective coding (integrating the categories into a core category) [29]. We did not use a priori codes nor did we apply a specific theoretical framework during any stage of the study. Rather, all themes, and hence the codes, emerged from the data. The qualitative software program MAXQDA was used to facilitate the analysis process. Besides looking for recurring themes, the researchers also actively searched for deviant cases. After analysing each of the first three interviews separately, MW and MD came together to discuss the codes and refine their understanding of the codes. After the third interview, the intercoder agreement as calculated with MAXQDA was 88%, which was considered sufficient to continue the coding process. The remaining interviews were equally divided among MW and MD and coded accordingly. After coding two or three interviews, MW and MW came together to discuss the codes. Any discrepancies were solved through discussion. During the coding process, MW and MD critically reflected and challenged themselves on assumptions about the respondents’ experiences, which sometimes led to adjustment of their interpretations. Additionally, all findings and interpretations were discussed with the other authors.

### Ethical considerations

Before participating in the interviews, oral and written information about the study aim and procedure was given to the participants, after which written consent was obtained from all participants. The oral and written information stated that participation was voluntary and that participants could withdraw themselves from the study at any moment without giving a reason. Participants were assured that their data were treated confidentially, and that information in the interviews that could identify them (such as names of hospitals, healthcare providers) would be anonymized in the transcripts. The need for ethical approval was waived by the Medical Ethics Committee of the VU University Medical Centre (reference 2015.033).

## Results

Characteristics of the 15 participants are shown in Table 1. The mean age of the respondents was 32.9 (range 25–42 years). Seven of the participants were primiparous, eight were multiparous. Twelve of the respondents were of Dutch ethnicity, three were of non-Dutch ethnicity (African, Asian and Turkish). All women finished at least intermediate vocational education, five also finished higher vocational education and four were university graduates. Three women received information based on the third trimester routine ultrasound that their baby might have growth restriction, for all other participants the third trimester routine ultrasounds did not show any abnormalities.

**Table 1:** Background characteristics of participants

Variables	
Age (years), Mean (range)	32.9 (25–42)
	N
Age group	
< 35 years	10
≥ 35 years	5
Parity	
Primiparous	7
Multiparous	8
Ethnicity	
Dutch	12
African	1
Asian	1
Turkish	1
Educational level	
Intermediate vocational education	6
Higher vocational education	5
University graduate	4
Ultrasound(s) indicated growth restriction	
Yes	3
No	12

While women expressed that the third trimester routine ultrasound did not do much for them in terms of relief and bonding with the baby, women in general expressed a strong need for a third trimester routine ultrasound scan. We will discuss this finding in more detail through three themes that emerged from the data analysis: (1) The third trimester ultrasound as a bonus (2) The third trimester ultrasound to get confirmation and (3) The third trimester ultrasound as part of a ‘normalisation process’, referring to the process of routine ultrasounds becoming a normal, unquestioned part of healthcare in pregnancy.

### The ultrasound as a bonus

Most women expressed that they felt much more confident about the health of their baby in the third trimester of their pregnancy than in the first two trimesters. Reasons mentioned were the fetal movements they experienced and the check-ups they had received from their midwife throughout their pregnancy. Women especially perceived the 20-week ultrasound as a turning point after which they could feel confident that everything would be fine. Most women mentioned fetal movements and ultrasound scans in the first two trimesters as being important for them feeling a bond with their baby; it made their pregnancy more real to them. Also, the increasing confidence in their baby’s health was important for them to allow themselves to feel connected to their baby. Compared to ultrasounds in the first two trimesters, women described the third trimester routine ultrasound as a far less exciting experience. Women did not feel much tension beforehand and although they were happy when everything turned out to be fine, none of the women experienced the kind of relief or strengthened bond they felt after the ultrasounds in the first two trimesters.

**[≥ 35 years, primiparous]** “*On our way to the 30-week ultrasound we were cycling and then I thought … I asked my boyfriend, are you nervous? And he said, no, actually I am not. And I said me not at all either, because you actually already know that everything is fine.*”

**[< 35 years, primiparous] “***I just feel confident now that the child is healthy and will remain healthy and at least … it’s moving a lot so eh.. Yeah, I did not find those ultrasounds that exciting. Just nice to see again and it gave a nice feeling, but not as exciting as the first ultrasounds. At that time I was more insecure if it would go well and now I have so much confidence that I do not feel that nervous about it.*”

Women generally described the third trimester routine ultrasound as a nice opportunity to see the baby one more time. Descriptions like ‘a bonus’, or ‘a luxury’ were given when women told how they experienced the third trimester routine ultrasound.

**[< 35 years, multiparous]** *“Then you just know what’s going on and.. yeah, without it … it’s maybe a luxury now, a very pleasant luxury.”*

**[< 35 years, multiparous]** *“Yeah, not really important. It is just something extra that you can get.”*

Although some couples went to every ultrasound together, women did not mention the need for their partner to attend the third trimester routine ultrasound ‘in case something would be shown’, as was the case with the ultrasound scans in the first two trimesters. Instead of their partner, some women took the third trimester routine ultrasound as an opportunity to bring other family members along, like their children or their parents.

**[< 35 years, primiparous]** *“I also liked that I could bring my family to show them briefly. At the 20-week scan I did not bring anyone else [apart from my partner] because I thought, well, we’ll do it together because all kinds of little things could show up of course. Then at the 30-week scan, I took my mother and my grandmother. So then they can already see it as well. It is still different than just the small picture and then they can see it move and hear the heartbeat … And at the second ultrasound [in the third trimester] I took my mother in law and my father, so that is like, gives it a little extra something.. at that ultrasound and that they are also involved a little bit more with the pregnancy.”*

Perceiving the third trimester routine ultrasound as less of a medical examination and more as an extra opportunity to see the baby for both themselves and their family sometimes led to disappointment for women when the ultrasound did not give a clear image of their baby, or when they felt that during the ultrasound not enough attention was paid to the social aspect of the procedure.

**[< 35 years, multiparous]** *“Yes, as if she did not have time for me actually, it felt like that. I also brought my mother in law that time, so it was actually very disappointing because I was looking forward to it. After that, we sat down and got an explanation and everything, but the ultrasound itself, I did not recognise a child on it. Only because she said: there is the nose and everything. Normally I see it much better but I don’t think she had an awful lot of time.”*

### The ultrasound to get confirmation

Around the time of the third trimester, women expressed they felt quite confident about the health of their baby and hardly perceived the third trimester ultrasound as a medical examination. However, most women reacted negatively to the idea of not getting any ultrasound anymore after 20 weeks gestation.

**[≥ 35 years, multiparous]** *“But from 20 [weeks] until giving birth is, I think, a very long period of nothing. I like it very much that they have another look, because on the 20-week ultrasound … it is only 20 weeks.”*

**[< 35 years, primiparous]** *“I was just talking about it with my midwife that normally after those 20 weeks, if you are not participating in the study, you don’t get any ultrasound anymore, nothing. And then I thought; that is a very long time. That basically, until it is born at 40 weeks, you don’t get an ultrasound anymore. So you don’t know how everything is going. I said; well, I actually don’t like that at all.”*

A need for confirmation was frequently expressed by women when they described their need for a third trimester routine ultrasound. As some women explained, judging the growth or general health of the baby either by feeling the baby move or by measuring growth by hand was a little vague. They got the feeling that this did not give enough information. Making the baby visible with an ultrasound made it less abstract and more real to them and easier to believe that the baby was really fine. Women expressed that they found it important for themselves and for other women to be able to get this confirmation and that it should become part of routine care. If not, they probably would have initiated to have some extra ultrasounds performed themselves.

**[< 35 years, multiparous]** “*You feel it move, so you know that it’s alive, but of course you don’t know if it’s really healthy and that kind of stuff.*”

**[< 35 years, primiparous]** *“No, I think they could make it part of the basic health insurance package that, indeed, you have one or two extra ultrasounds. Yeah I think like.. I personally think that it reassures a lot of women. At least for me it did, because if I had not taken part in this study I might just have done another two or three ultrasounds. Just to see for myself if it was okay.”*

A few women specifically mentioned that they wanted to know if their baby was growing well, or was not getting too big. Most women, however, did not have specific concerns about their baby. They expressed a general need to see on the ultrasound that everything was fine.

**[≥ 35 years, primiparous]** *“Not that you are worried, but at the moment that you see it and get the confirmation that everything is fine and everything goes according to plan, that is nice.”*

**[≥ 35 years, multiparous]** “*I realize that it gives me a good feeling every time when I hear that it’s fine.*”

The three women whose babies seemed small according to the third trimester ultrasound and who were referred to the hospital for further ultrasounds, expressed that the result of the routine ultrasound scan, and getting subsequent ultrasounds in the hospital were stressful for them. Also, they were aware that these stressful feelings might be unnecessary if the baby turned out to be not growth-restricted according to subsequent ultrasound scans, or at birth. On the other hand, they were glad that everything was checked carefully. The idea that something could be wrong that would have been missed otherwise, seemed to be a strong motivation for them to endorse the third trimester routine ultrasound. For one of these women, the ultrasounds in the hospital indeed showed that her baby was not growth-restricted. Nevertheless, she looked back positively on the third trimester routine ultrasounds, because the idea that everything was checked made her feel good.

**[< 35 years, multiparous]** “*And suppose there is something wrong, then you are happy yes. That you … eventually I am happy with it I guess. Because everything has been checked properly after all. In principle, you don’t really have any other ultrasounds at the end, unless there is a reason for it, I think? So maybe it is a good idea to do it anyway*.”

Of all the women interviewed, only two women said that they did not need a routine ultrasound in the third trimester. One of these women explained that she had confidence that the routine visits at her midwife were sufficient to detect if anything was wrong **[< 35 years, multiparous]**. The other woman said that she had not expected any ultrasound in the third trimester and that she was just happily surprised to get one. She would not really mind it, however, if she would not have had it **[< 35 years, multiparous]**.

### The ultrasound as part of a normalisation process

When trying to explain the origins of their need for confirmation by the third trimester ultrasound, women frequently mentioned that it would seem strange to them to not get any ultrasound after 20 weeks pregnancy. Getting ultrasounds was something they got used to during their current or previous pregnancy. Imagining that they would not get a routine ultrasound in their third trimester while they frequently had routine ultrasounds in the first two trimesters, or wile they got a third trimester routine ultrasound scan in a previous pregnancy, was difficult for them.

**[≥ 35 years, primiparous]** *“But it would seem very strange to me if I would not have it in another pregnancy. While at the same time it is not.. yeah, because if I say it like that, it would seem like a check-up or something, while I was not nervous about it. But it just seems very weird to me not to have it. So maybe it is because I got used to it and don’t know any better because I am getting this already since the beginning, from those 15 weeks. So maybe I just got used to it. But I think it is nice for women to have extra ultrasounds. But yeah.. then it sounds like, well, I don’t really know.”*

**[≥ 35 years, multiparous]** *“With my first daughter, I also participated in a study, so at that time I also had it. The idea that I would not get anything from 20 weeks till the birth, I would find that very minimal.”*

Some women used the word ‘milestone’ when they described the routine ultrasounds. When they had received one, they were soon looking forward to the next one. Getting confirmation from the routine ultrasound made them feel good, however, after a while that feeling faded and they were longing for another ultrasound. This way, they were living from one ultrasound to the next.

**[< 35 years, primiparous]** *“You really look forward to the next time you can see her, this a girl like … , that you can see her again on the screen let’s say. That you see the little heart beating at least and that you get the confirmation like … , well, it is all fine. And well, that is just a very nice feeling.”*

**[< 35 years, primiparous]** *“I always look forward to it. Or at least I always like it very much and I am counting down every time yes.”*

In general, women saw routine ultrasounds as an integral part of pregnancy. In other words, ultrasounds seemed to be ‘normal’ for most women. Accordingly, women did not spend much time thinking about the option of extra ultrasounds in the third trimester. It was just a reasonable thing to do for most women, a chance that you take without questioning. One woman expressed for example: *“The more ultrasounds the better”* **[< 35 years, multiparous]**. And another woman said: *“Yeah, if you can get a free ultrasound, you take that chance”* **[< 35 years, primiparous]**. Another woman told that before her first pregnancy, she got the impression from movies that every time you go for a check-up, you get an ultrasound and that she and her partner were surprised that in reality that was not the case. When they got the chance to see their baby two more times, they directly took that chance **[< 35 years, multiparous]**. One woman was the exception. She was afraid that ultrasounds might be harmful for the baby and therefore had some doubts whether she should accept the third trimester routine ultrasounds, but in the end she found it important to get the confirmation about the health of the baby **[< 35 years, primiparous]**. There were also women, however, who did not hesitate to take the third trimester ultrasound, but who also expressed some concerns. They felt that all the information available nowadays can take away worries on the one hand, but also emphasises risks concerning pregnancy which can cause women to feel insecure.

**[≥ 35 years, multiparous]** *“I have very mixed feelings about it. On the one hand it is nice what they can see and what they can check. On the other hand, it can also bring many uncertainties.”*

**[< 35 years, primiparous]** *“That is the case nowadays.. I think we are driving each other crazy with all these “oh, is yours that small”.. and everyone knows best and you should have a camera on your baby monitor and you need an Aerosleep matrass and.. ”*

However, they found it difficult not to go along with additional routine ultrasounds while other people in their environment did, and while the current technology offers you these opportunities.

**[< 35 years, primiparous]** *“But yeah, you also do not want to not do it, because then you are the only one who does not have it, so that would then be wrong again probably. I think that if we all just act normally, then that is just fine. [..] And that’s just terrible but, you do want the best for your child, don’t you? And I just feel like.. they just play on your feelings. And you don’t want to fall behind or do less well for you child. So yes, then you just give in in the end.”*

## Discussion

In this qualitative study we explored the experiences of pregnant women with third trimester routine ultrasounds, and identified three themes emerging from the interviews. The first theme concerned the perception of *the third trimester routine ultrasound as a bonus*. Women were generally confident about their baby’s health in the third trimester and seemed to perceive the third trimester routine ultrasound as a nice opportunity to see their baby one more time, alone, or in the presence of their partner or family. Despite this perception of the third trimester as more of a fun and social event than a medical examination, most women expressed a need to get confirmation from a third trimester routine ultrasound that their baby was fine. This was expressed in the second theme: *The third trimester routine ultrasound to get confirmation*. The contradiction between perceiving the ultrasound as a bonus on the one hand, but still feeling the need for confirmation on the other hand, might be related to the third theme we identified, *the ultrasound as part of a normalisation process*; getting several routine ultrasounds throughout pregnancy made the ultrasound become an integral part of pregnancy for women, something one accepts without questioning.

The first theme, *the third trimester routine ultrasound as a bonus*, showed that the third trimester routine ultrasound plays a different role for women than the routine ultrasounds in the first two trimesters. As we expected, reassurance and bonding with the baby was experienced less in the third trimester since the fetal movement and check-ups that took place earlier in pregnancy already made women feel confident and connected with their baby. This implies that the claim about the psychological benefits of routine ultrasounds regarding reassurance and bonding, which is supported by several studies [1, 30, 31], might not apply to the third trimester routine ultrasound. Instead, the perception of the third trimester routine ultrasound seemed to be more similar to a ‘keepsake’ ultrasound; it is less valued for its diagnostic values and more for its entertaining properties.

Although women perceived the third trimester routine ultrasound as a bonus rather than a medical event, most women nevertheless expected to have a routine ultrasound in the third trimester. This was illustrated by our second theme: *The third trimester routine ultrasound to get confirmation.* The idea of not getting a routine ultrasound in the third trimester seemed to make women uncomfortable. Most women, however, did not state a specific concern when they expressed this need. They seemed to feel a general need for a routine ultrasound that confirmed that everything was fine. This was also shown by a study from Thomas et al. [24] who interviewed women about their experiences with ultrasound scans during all trimesters of pregnancy. While women in the first trimesters seemed to be concerned about whether their baby was viable and healthy, women in the third trimester used much more general terms to express that they needed to know their baby was fine.

Perhaps the most intriguing result from our study was the discrepancy between the perception of the third trimester routine ultrasound as a bonus on the one hand, and the expressed need to get confirmation on the other hand. This discrepancy might be related to the third theme that emerged from our data: *The ultrasound as a normalisation process*. Women in our study had become used to regular ultrasounds throughout the first and second trimester of pregnancy and therefore most women also wanted a routine ultrasound in their third trimester. Simply because it just felt weird not getting one. Related to this, almost all women in our study indicated that they did not need time to consider the option of extra ultrasounds in the third trimester and immediately took the chance. Even the few women who expressed to have some doubts about whether the third trimester routine ultrasound was of added value, did not consider to decline the ultrasound. This concept of normalisation was also found by Edvardsson et al. [10] who held focus groups with 37 Australian midwives about their views on the use of ultrasounds during pregnancy. According to these midwives, ultrasounds have become accepted to be part of pregnancy and are generally unquestioned. Not only by the antenatal health care system, but also by the society. The midwives further observed that declining an ultrasound in this kind of society is difficult because it is hard to deviate from the existing norms, and women might feel they will be blamed to have acted as irresponsible if they do not use the available technology. This social pressure experienced by pregnant women to make certain choices regarding prenatal testing in a technology-driven society was already described in 1993 by Gregg [32].

Concerning the practical implications of our results, one might argue that our findings show that as far as psychological consequences are concerned, it cannot hurt to offer women a routine ultrasound in the third trimester since most women do not seem to experience much stress beforehand and express a need for confirmation. On the other hand, it is important to look at the reasons why pregnant women need confirmation from the third trimester routine ultrasound. As our results showed, the need to get confirmation does not seem to originate from a real concern, but rather seems to be driven by normalisation. Apart from the question whether women are still having a real choice in this case, or are acting according to what they consider to be standard care, this raises some ethical questions. Firstly, by introducing a third trimester routine ultrasound while women actually express they already know their baby is fine, one might create a ‘medicalised’ antenatal care system where it will be difficult for women to be able to rely on their own sense of wellbeing [24]. Secondly, normalisation of routine ultrasounds carries with it the danger of creating a gap between women’s expectations of the routine ultrasounds, and the actual purpose of the routine ultrasound, including its possible outcomes and limitations [1, 31, 33]. Consequently, women might falsely assume everything will be fine, or be unprepared for adverse findings [1]. As was also discussed by Edvardsson et al. [10], this gap between expectations and true purpose endangers the fundament of informed consent; patients decide to undergo a medical procedure without a full understanding of it. Thirdly, research addressing the effectiveness of the third trimester routine ultrasound has as yet been inconclusive [9]. Letting society finance third trimester routine ultrasounds because of pregnant women’s need for confirmation while gains in terms of pregnancy outcomes are unclear, seems hard to defend in a healthcare system that is getting more expensive and where prioritisation is required [34].

For future studies that address the psychological impact of a third trimester routine ultrasound, it would be interesting to interview women before as well as after their third trimester routine ultrasound. It might be that their perception of how they felt before the ultrasound is coloured by the result of the third trimester routine ultrasound. For example, if women get the confirmation that everything is fine, they might interpret the stress they felt beforehand as lower than it was. Related to this, it would also be interesting to interview women about their experiences with the third trimester routine ultrasound after giving birth. It might be that when women find out that the growth-estimation of the third trimester routine ultrasound differs from the actual size, it will change their perception. To get a complete picture of the psychological impact of the third trimester routine ultrasound, it is important to take all these phases into account. Additionally, interviewing women who declined a third trimester routine ultrasound, might give us a more complete picture of the impact of offering third trimester routine ultrasounds.

### Strengths and limitations

By using a qualitative approach, we were able to provide insight in low-risk pregnant women’s experiences with third trimester routine ultrasounds at a thorough level. Effort to give a representative view of experiences was made by interviewing women with different background characteristics, and conducting interviews until no new information emerged. Our procedure of independently coding the interviews and discussing the interpretations, while carefully looking for deviant findings, further enhanced the validity of our findings. A limitation of our study was that we only interviewed women who were able to speak Dutch. Since women who are not able to speak Dutch might show less resemblance to our included population, this could limit the generalizability of our results. Another possible limitation related to the generalizability of our study might be that women who declined to be interviewed might have differed in their responses from the included sample. However, as we were able to include women with a variety of background characteristics (parity, age, ethnicity and educational level), we do believe we were able to represent the experiences of the majority of low-risk pregnant women’s experiences with third trimester routine ultrasounds.

## Conclusion

This study highlights the importance of looking critically at the psychological impact and gains of the third trimester routine ultrasound for pregnant women. While women generally hold positive views about getting a third trimester routine ultrasound, this ultrasound might not provide the same benefits regarding reassurance or bonding as routine ultrasounds in the first two trimesters. Moreover, this study showed that the expressed need of pregnant women for a routine ultrasound in the third trimester may arise from the normalisation process regarding routine ultrasounds. This finding raises questions about whether the third trimester routine will contribute positively to the psychological well-being of pregnant women, or mainly to a further dependence on routine ultrasounds. We recommend to examine the psychological impact of third trimester routine ultrasounds in larger future studies. Together with further research on the clinical effectiveness of third trimester routine ultrasounds, these results should be taken into consideration when balancing the gains of introducing a third trimester routine ultrasound against unwanted side effects and costs.

## Data Availability

Quotes from the interviews are published in this article. Full transcripts are not publicly available as they are written in Dutch, but are available from the corresponding author on reasonable request.

## Appendix

**Table 2:** Topic-list

*Feelings during pregnancy* • Could you describe the feelings you experience or have experienced during pregnancy? • Are there any factors (events, situations) that evoked positive or negative feelings? • Did you experience changes in the feelings that you experienced during pregnancy? What caused these changes? • Do you ever feel worried during your pregnancy about the pregnancy itself, or giving birth? • Are there any factors (events, situations) that made you feel worried? • Did you experience changes in the extent to which you felt worried? What caused these changes? • To which extent does your baby occupy your thoughts? • When you think about your baby, what kind of thoughts and feelings does this bring to your mind? • Could you describe to which extent these thoughts and feelings have changed during your pregnancy?	
*Experiences with a third trimester routine ultrasound* • How did you generally experience receiving ultrasounds? • How did you experience receiving a third trimester routine ultrasound compared to other ultrasounds? • What aspects of the third trimester routine ultrasound did you experience as positive, or less positive? This may concern the preparation, the ultrasound itself, or the consult afterwards. • Who accompanied you during the third trimester routine ultrasounds?*	
*The possible influence of a third trimester routine ultrasound on feelings experienced during pregnancy* • Did the third trimester change your feelings concerning your pregnancy? • If so, to what extent did the third trimester routine ultrasound affect your worries about your pregnancy? • To what extent did the third trimester routine ultrasound change your feelings about your baby? • Imagine that you would not have been offered third trimester routine ultrasounds, how would that have affected you?*	

* These questions were added to the topic-list after the first three interviews
